# Les maladies inflammatoires chroniques de l’intestin: que se passe-t-il quand le SARS-CoV-2 arrive? Résultats préliminaires du Centre Hospitalier Universitaire Hassan II de Fès, Maroc (à propos d’un cas)

**DOI:** 10.11604/pamj.2021.38.382.27235

**Published:** 2021-04-19

**Authors:** Hakima Abid, Imane Atmani, Nada Lahmidani, Mounia El Yousfi, Dafr-Allah Benajah, Sidi Adil Ibrahimi, Mohamed El Abkari

**Affiliations:** 1 Service d’Hépato-Gastro-Entérologie, Centre Hospitalier Universitaire Hassan II, Faculté de Médecine et de Pharmacie de Fès, Université Sidi Mohamed Ben Abdellah, Fès, Maroc

**Keywords:** Maladies inflammatoires chroniques de l´intestin, maladie de Crohn, rectocolite hémorragique, COVID-19, à propos d’un cas, Chronic IBD, Crohn’s disease, ulcerative colitis, COVID-19, case report

## Abstract

L´infection par le SARS-CoV-2 représente une grande source de préoccupation et une nouvelle menace pour les patients immunodéprimés. Les patients atteints de maladies inflammatoires chroniques de l´intestin (MICI) courent un risque accru d'infection, en particulier lorsqu'ils ont une maladie active et prennent un traitement immunosuppresseur. Ce travail vise à évaluer les caractéristiques cliniques, biologiques et radiologiques ainsi que la gestion et les résultats du COVID-19 chez trois malades présentant une MICI, recrutés sur une période de 3 mois au sein du Centre Hospitalier Universitaire Hassan II Fès, Maroc. Tous les malades étaient en poussée de la maladie. Tous avaient des symptômes légers ou étaient asymptomatiques. Aucun changement ou retard dans leurs schémas thérapeutiques n'est survenu, et aucun n'a développé un COVID-19 sévère. Les résultats du test de transcriptase inverse-réaction en chaîne par polymérase (RT-PCR) étaient positifs chez tous les patients. Radiologiquement, nous avons retrouvé dans un cas des opacités en verre dépoli sur le scanner thoracique. Le traitement était basé sur l'association d'hydroxychloroquine et d'azithromycine. L'évolution sous traitement était bonne pour tous les patients. Ce rapport préliminaire suggère que les malades présentant une MICI ne courent peut-être pas un risque plus élevé de développer le COVID-19 par rapport à la population générale.

## Abstract (en)

SARS-CoV-2 infection is a major concern and a new threat to immunocompromised patients. Patients with chronic inflammatory bowel diseases (IBDs) are at increased risk of infections, in particular when they have active disease and are on immunosuppressive treatment. The purpose of this study was to assess the clinical, biological and radiological features of three patients with COVID-19 associated with chronic IBD as well as their management and outcomes. The study was conducted at the Hassan II University Teaching Hospital in Fes, Morocco over a 3-month period. We assessed all patients with disease onset. All patients had mild symptoms or were asymptomatic. No changes or delays in treatment regimens occurred and none of patients developed severe COVID-19. Reverse transcription polymerase chain reaction (RT-PCR) test results were positive in all patients. Radiological examinations were conducted. Chest scanner showed ground-glass opacities in one case. Treatment was based on hydroxychloroquine with azithromycin. Outcome was good in all cases. This preliminary report suggests that patients with chronic IBD aren’t at higher risk of developing COVID-19 compared to the general population.

## Introduction

L'Organisation Mondiale de la Santé (OMS) a récemment déclaré l'épidémie de maladie à un nouveau coronavirus (SARS-CoV-2) de 2019 comme une pandémie de préoccupation internationale le 11 mars 2020 [1]. Au Maroc le premier cas de COVID-19 a été identifié le 2 mars 2020, avec une augmentation progressive pour atteindre 190416 cas le 23 octobre 2020 [2]. Les maladies inflammatoires chroniques de l´intestin (MICI) affectent les personnes de tous les âges. Leur prise en charge consiste à traiter une inflammation incontrôlée nécessitant des immunosuppresseurs et/ou de la biothérapie [3]. Cependant, ces thérapies, en plus de la malnutrition qui peut compliquer les MICI, peut affaiblir le système immunitaire et mettent potentiellement ces patients à un risque accru d'infections et de complications infectieuses [4]. Par conséquent, il est à craindre que les patients atteints de MICI soient plus à risque de développer le COVID-19 et ont un risque accru d'évolution vers une forme clinique plus sévère par rapport à la population générale. Afin d´analyser la particularité de la maladie COVID-19 chez les malades atteints de MICI, nous avons mené une étude prospective descriptive analytique du 01/08/2020 au 31/10/2020 au sein du CHU Hassan II de Fès au Maroc. Les informations démographiques et toutes les caractéristiques, y compris l'exposition, les antécédents médicaux, les symptômes, les résultats de l´imagerie et les analyses biologiques, les traitements et l'évolution de chaque patient ont été obtenues à partir du système Hosix (dossiers médicaux électroniques) du CHU Hassan II Fès, Maroc.

## Patient et observation

Entre le premier août 2020 et le 31 octobre 2020, au sein du CHU Hassan II de FES, nous avons recruté 3 patients consécutifs atteints de MICI avec un diagnostic de COVID-19. Deux malades avaient une maladie de Crohn (MC) et un malade avait une rectocolite hémorragique (RCH). Les caractéristiques de base des patients sont présentées dans le Tableau 1. Tous nos malades étaient identifiés suite à un contact étroit avec un cas positif au cours de leur hospitalisation pour une poussée de MICI. Le diagnostic du COVID-19 a été confirmé chez tous nos patients avec des résultats positifs de la RT-PCR sur un échantillon d'écouvillon nasopharyngé. Dans tous les cas, les valeurs de la protéine C-réactive (CRP), des globules blancs, des fonctions rénale et hépatique étaient normales. Radiologiquement, le scanner thoracique avait objectivé une atteinte parenchymateuse en verre dépoli minime estimé à 10% évoquant une pneumonie à SARS-CoV-2 dans un cas, alors qu´il était normal dans les 2 autres cas.

**Tableau 1:** caractéristiques de base des patients atteints de MICI avec COVID-19

	Total (n=3)	MC (n=2)	RRCH (n=1)
Age en années (intervalle)	40 [28-63]	28-63	29
Sexe			
Fèminin	1	1	-
Masculin	2	1	1
Types de comorbiditès			
Tabagisme	0	0	0
Hypertension artérielle (HTA)	0	0	0
Diabète	0	0	0
Obèsitè	0	0	0
Maladie cardiorespiratoire	0	0	0
Spondylarthrite ankylosante	1	1	0
Activitè de la maladie			
Rèmission	0	0	0
Active	3	2	1
Traitements concomitant des MICI			
6 mercaptopurine	3	2	1
Corticostèroïdes oraux	3	2	1
Anti-TNF	2	1	1
Les symptômes liés au COVID-19			
Fièvre	0	0	0
Toux	1	1	0
Dysosmie ou dysgueusie	0	0	0
Arthralgie ou myalgie	0	0	0
Dyspnée	0	0	0

Tous nos patients ont été hospitalisés et traités avec des contrôles réguliers. La saturation en oxygène était comprise entre 92 et 95% à l´air ambiant. Les modalités de traitement étaient axées sur un soutien symptomatique et spécifique. Le traitement symptomatique était basé sur la vitamine C, le zinc et le paracétamol en cas de fièvre. Nous avons adopté, suite aux recommandations ministérielles nationales marocaines, une thérapie à base d'hydroxychloroquine précédée d'un électrocardiogramme à la recherche de troubles du rythme cardiaque. Ainsi, Ils ont reçu une bithérapie à base d'hydroxychloroquine pendant 10 jours, d'azithromycine pendant 7 jours et l'héparine de bas poids moléculaire à dose préventive. La réponse au traitement a été bonne avec une PCR de contrôle négatif après 15 jours. Aucun effet secondaire n'a été noté jusqu'au 31 octobre 2020. Concernant le traitement de la MICI sous-jacente, la corticothérapie orale a été maintenue, alors qu´on avait repris les immunosuppresseurs après résolution de la maladie COVID-19.

## Discussion

La pandémie liée au SARS-CoV-2 est une maladie infectieuse qui s´est propagée initialement dans la province de Wuhan en chine; puis elle a été déclarée comme une pandémie mondiale. Cette pandémie conditionne certainement la stratégie de traitement des MICI, puisqu'il est pertinent de penser que le risque infectieux augmente par rapport à la population générale en raison d'une détérioration générale du système immunitaire typique des maladies auto-immunes combiné à l'effet iatrogène généré par l'utilisation de corticostéroïdes et de médicaments immunosuppresseurs [5]. Durant la période de notre étude étalée sur trois mois, on a diagnostiqué l´infection au SARS-CoV-2 chez trois malades ayant une MICI parmi 233 malades atteints de COVID-19 au cours de cette période soit un taux de 1,28% (mise à jour 23/10/2020) ce qui rejoint les résultats d´une série faite en Italie dont seulement 13 cas de MICI COVID-19 signalés parmi plus de 105000 cas de COVID-19 dans la population générale (statistiques déclarées jusqu´à avril 2020) [6]. Aux États-Unis et en Europe, seulement 239 cas ont été rapportés chez des patients atteints de MICI en association avec la maladie COVID-19 (sur un total 205000 cas aux États-Unis et 446000 en Europe) jusqu´en avril 2020 [6]. En Espagne, le premier cas de COVID-19 a été détecté le 31 janvier 2020. Depuis lors, un total de 220325 cas a été diagnostiqué, dont 959 cas détectés chez les patients atteints d'une MICI (mise à jour 05/05/2020) [7]. En outre, les rapports initiaux de la Chine et du nord de l'Italie suggéraient un taux d'infection plus faible chez les patients atteints de MICI par rapport à la population générale. Les patients atteints de MICI étaient sous-représentés parmi ceux diagnostiqués avec une maladie symptomatique du COVID-19 dans les deux localités. Il est possible que les patients atteints de MICI associée à la COVID-19 soient sous-évalués et sous-déclarés par rapport aux personnes sans MICI [6].

L´âge moyen de nos malades était de 40 ans. On avait noté une prédominance masculine malgré le nombre réduit de nos malades, ce qui rejoint la littérature. Une étude faite dans un centre médical régional des MICI à l´hôpital Renmin de l'Université de Wuhan en Chine sur 318 patients atteints de MICI diagnostiqués COVID-19. L'âge médian était de 39 ans, avec une prédominance masculine soit 61.3% [8]. Une autre étude faite par un groupe italien pour l'étude des MICI (IG-IBD) chez 79 malades avec un diagnostic de COVID-19, a rapporté que l´âge médian était de 49 ans, avec prédominance masculine soit 55% [9]. Par ailleurs, il faut également tenir compte du fait que les patients atteints de MICI ont tendance à être plus jeunes et ont moins de comorbidités associées que la population générale [10]. L'âge avancé et la présence de comorbidités sont en eux-mêmes les facteurs de risque les plus importants d'une mauvaise évolution de la maladie COVID-19 [11]. C´est ainsi que dans la série de Wuhan 15.4% des patients avaient des comorbidités notamment l´hypertension artérielle (8.8%), le diabète (1.6%), les maladies cardio-respiratoires (1.3%) et le tabagisme (2.5%) [8]. La série Italienne rapporte elle aussi la présence de comorbidités chez 38% des malades ayant une MICI et diagnostiqués COVID-19+, ainsi on avait noté une hypertension artérielle chez 11% des cas, une maladie coronarienne chez 6%, une maladie respiratoire chez 6% ainsi qu´une spondylarthrite ankylosante chez 3% des cas [9]. Nos malades ne présentaient pas d´intoxication tabagique ni de comorbidités associés mise à part spondylarthrite ankylosante chez un malade.

Durant notre étude, on avait rapporté 2 cas de MC et un cas de RCH. Tous nos malades étaient en poussée de leur maladie. Sur une série de Wuhan, 59% des malades étaient en rémission parmi un totale de 318 MICI (114 cas de MC et 204 cas de RCH) [8]. Dans une large cohorte de patients (2000 cas) atteints de MICI en France (Hôpital universitaire de Nancy) et d'Italie (Humanitas à Milan) de 4000 patients, 15 patients COVID-19 positifs sont rapportées: neuf patients avaient une MC dont 3 avaient une maladie active et la majorité était sous biothérapie et/ou immunosuppresseurs au moment du diagnostic de l'infection à COVID-19 [12]. Dans la série Italienne, 12% des MC et 35% de ceux atteints d´une RCH étaient en poussée [9]. Concernant les résultats de notre étude, nous avons 2 malades asymptomatiques, et un seul cas qui a présenté une forme bénigne de la maladie (toux sèche). Cependant, les données de la série italienne rapportent que les symptômes les plus courants du COVID-19 chez les malades MICI étaient sous forme de fièvre (90%), toux sèche (66%), dysosmie et/ou dysgueusie (24%), arthralgies/myalgies (23%), dyspnée (19%), diarrhée (15%) et de rhinorrhées (16%) [9]. Le diagnostic de COVID-19 a été confirmé chez tous nos malades par RT-PCR sur des prélèvements nasopharyngés. Dans l´étude de l´Italie, le diagnostic a été confirmé par RT-PCR sur un prélèvement nasopharyngé chez 63% des patients tandis que 37% cas ont été confirmés par des signes cliniques et radiologiques [9]. La tomodensitométrie thoracique est un outil de diagnostic très sensible pour détecter la pneumonie et la sensibilité au COVID-19 serait de 97,5% [12]. Dans notre série, nous avions 1 seul patient qui avait des lésions minimes en verre dépoli avec atteinte estimée à 10%, ce patient avait une toux sèche sans dyspnée.

La COVID-19 ne semble pas être plus grave chez les patients atteints de MICI par rapport à la population générale, même chez les patients sous traitement immunosuppresseur [13,14]. Dans le registre multicentrique SECURE-IBD, 959 patients ont été rapportés (dont 168 en Espagne): 33% nécessitent une hospitalisation, 6% sont admis aux soins intensifs, 5% nécessitent une respiration assistée avec une mortalité de 4% (mise à jour le 05/05/2020) [15]. La série italienne montre que les patients atteints de MICI active nécessitent plus d'hospitalisation et de ventilation assistée que les patients atteints de MICI en rémission [9]. Chez nous, les malades étaient hospitalisés dans un service médical sans recours à la ventilation artificielle et aucun décès n´a été déploré. Selon le protocole thérapeutique ministériel national Marocain, nous avons mis les malades sous l'association hydroxychloroquine et azithromycine. L'hydroxychloroquine, un médicament antipaludique, connu pour ses effets anti-inflammatoires et antiviraux, à faible coût et facilement disponible, est une pratique prometteuse pour traiter l'infection au COVID-19 dans des conditions de gestion raisonnables. L'une des principales études françaises a suggéré que l'hydroxychloroquine, en association avec l'antibiotique azithromycine, pourrait fonctionner comme traitement du COVID-19 [16]. Les résultats de cette étude ne devraient pas être concluants compte tenu de sa conception d'observation. Selon une étude plus récente, l´hydroxychloroquine n´est pas associée à une réduction de la mortalité à 28 jours. En revanche, elle est associée à une augmentation de la durée d´hospitalisation et à un risque accru d´évolution vers une ventilation mécanique invasive ou le décès [17].

Plusieurs études ont examiné le rôle de la supplémentation en vitamines pour contrôler la réplication virale [18]. Malgré le manque d'essais contrôlés randomisés dans ce scénario, nous avons maintenu nos patients atteints de MICI-COVID-19 sous vitamine C et zinc. Ce qui concerne le traitement de la MICI, la corticothérapie par voie orale était maintenu, cependant, les anti-TNF (tumor necrosis factors) et les immunosuppresseurs ont été interrompus. En général, un patient atteint de MICI doit être traité avec les mêmes thérapies que celles choisies en dehors de la pandémie COVID-19. Chez un patient qui présente une MICI qui a été testé positif pour le SARS-CoV-2 mais asymptomatique et dont le traitement a été interrompu, les médicaments contre les MICI peuvent être repris après 14 jours [19]. En cas d'apparition de signes et de symptômes évocateurs du COVID-19, il est recommandé de suspendre le traitement immunosuppresseur jusqu'à la résolution de l'infection. Le traitement peut être repris après résolution complète des symptômes du COVID-19 ou, idéalement, après deux tests PCR négatifs de l'écouvillon nasopharyngé, prélevés à plus de 24 h d'intervalle [19].

## Conclusion

Il était raisonnable de penser que l'infection par le SARS-CoV-2 chez les patients atteints de MICI soit plus grave, en particulier chez les patients sous médicaments immunosuppresseurs. Cependant, nous avons constaté que les patients atteints de MICI n'ont pas un risque accru de développer une infection au COVID-19, ni de développer des formes sévères lorsqu'ils sont infectés. La plupart d'entre eux ont présenté des résultats cliniques favorables, suggérant un effet potentiel «Protecteur» de la thérapie anti-inflammatoire chez ces patients, sans pouvoir établir un véritable lien de causalité. Cependant, notre échantillon reste limité ne pouvant pas nous permettre de tirer des conclusions finales. Ainsi, notre travail continue pour avoir des données plus crédibles.
